# Challenges in microarray class discovery: a comprehensive examination of normalization, gene selection and clustering

**DOI:** 10.1186/1471-2105-11-503

**Published:** 2010-10-11

**Authors:** Eva Freyhult, Mattias Landfors, Jenny Önskog, Torgeir R Hvidsten, Patrik Rydén

**Affiliations:** 1 Department of Clinical Microbiology, Division of Clinical Bacteriology, Umeå University, 901 85 Umeå, Sweden; 2 Computational Life Science Cluster (CLiC), Umeå University, 901 87 Umeå, Sweden; 3 Department of Mathematics and Mathematical Statistics, Umeå University, 901 87 Umeå, Sweden; 4 Department of Statistics, Umeå University, 901 87 Umeå, Sweden; 5 Umeå Plant Science Centre, Department of Plant Physiology, Umeå University, 901 87 Umeå, Sweden

## Abstract

**Background:**

Cluster analysis, and in particular hierarchical clustering, is widely used to extract information from gene expression data. The aim is to discover new classes, or sub-classes, of either individuals or genes. Performing a cluster analysis commonly involve decisions on how to; handle missing values, standardize the data and select genes. In addition, pre-processing, involving various types of filtration and normalization procedures, can have an effect on the ability to discover biologically relevant classes. Here we consider cluster analysis in a broad sense and perform a comprehensive evaluation that covers several aspects of cluster analyses, including normalization.

**Result:**

We evaluated 2780 cluster analysis methods on seven publicly available 2-channel microarray data sets with common reference designs. Each cluster analysis method differed in data normalization (5 normalizations were considered), missing value imputation (2), standardization of data (2), gene selection (19) or clustering method (11). The cluster analyses are evaluated using known classes, such as cancer types, and the adjusted Rand index. The performances of the different analyses vary between the data sets and it is difficult to give general recommendations. However, normalization, gene selection and clustering method are all variables that have a significant impact on the performance. In particular, gene selection is important and it is generally necessary to include a relatively large number of genes in order to get good performance. Selecting genes with high standard deviation or using principal component analysis are shown to be the preferred gene selection methods. Hierarchical clustering using Ward's method, k-means clustering and Mclust are the clustering methods considered in this paper that achieves the highest adjusted Rand. Normalization can have a significant positive impact on the ability to cluster individuals, and there are indications that background correction is preferable, in particular if the gene selection is successful. However, this is an area that needs to be studied further in order to draw any general conclusions.

**Conclusions:**

The choice of cluster analysis, and in particular gene selection, has a large impact on the ability to cluster individuals correctly based on expression profiles. Normalization has a positive effect, but the relative performance of different normalizations is an area that needs more research. In summary, although clustering, gene selection and normalization are considered standard methods in bioinformatics, our comprehensive analysis shows that selecting the right methods, and the right combinations of methods, is far from trivial and that much is still unexplored in what is considered to be the most basic analysis of genomic data.

## Background

Cluster analysis is a common approach to examine microarray expression data used both to group genes and samples/individuals. As an unsupervised method, the main advantage of cluster analysis is the ability to compare the expression profiles of different samples and detect groups of samples with similar expression profiles, e.g. to separate cancer patients likely to develop metastases without treatment from patients who are not likely to develop metastases and hence would not benefit from treatment. However, cluster analysis is by some believed to be overused [1] and is in need of thorough evaluation.

A few studies have evaluated different clustering methods and similarity metrics on real-world microarray data. One study found that model-based clustering (e.g. Mclust) and k-means performed best on cancer data, and that the frequently used hierarchical clustering method performed poorly [2]. Two other studies also report model-based clustering as one of the best choice for gene clustering [3,4], while yet another study found that performance varied too much between different evaluation criteria to be able to decide on one best method [5]. As has been the case in other bioinformatics areas, consensus methods have been shown to outperform the single best clustering method [6]. Although some agreement can be found in these studies, such as a tendency to rank model-based clustering highly and hierarchical clustering lowly, there are even more disagreements. One reason might be that these comparative studies did not consider many of the other analysis choices that inevitably have to be made in a complete microarray experiment. Both pre-processing of microarray data (normalization, missing value imputation, standardization) and gene (feature) selection is almost always performed before clustering and the choice of these methods can have dramatic effects on the final clustering results. Previous studies have analyzed the effect of normalization on gene clustering, showing that the non-linear loess normalization outperforms simpler normalizations such as scale and location normalizations [7]. Strengths and weaknesses of different imputation approaches have also been evaluated [8]. Maybe most importantly, the strong effect of gene selection on cluster analysis (mainly gene clustering) has been demonstrated using simulated data [9]. The importance of gene selection is not surprising given the impossibility of successful classification when discriminatory genes make up a small fraction of the total number of genes considered [10].

In this study we have used seven cancer data sets to demonstrate how cluster analysis is affected by the consecutive sub-processes; normalization of the microarray data, imputation of missing values, gene selection, standardization and clustering. To the best of our knowledge, no comparative study of clustering methods has taken into account all these accompanying analysis choices when evaluating the relative ability of these methods to retrieve natural groupings in gene expression data. We have focused on clustering of samples (individuals) commonly used in microarray studies to, e.g., identify subtypes of cancer with unique properties such as response to treatment. The analyses are implemented in R [11].

We show that the variation between data sets is huge and the best way to analyze one data set might not be optimal for another. However, some general conclusions can be drawn. We show that gene selection, clustering method and to some extent also normalization are important to the performance, and the choice of missing value imputations and standardization has only a minor effect on the performance (at least for the type of design considered in this study).

## Methods

### Data

Seven previously published data sets are included in this study. All the data sets describe 2-channel microarray experiments with a common reference design [12] involving various types of human cancer. All experiments are published in high impact journals and are relative large, containing data from 40 to 133 samples. Two of the experiments used commercially produced arrays (Agilent) while the other experiments used custom made arrays. One-channel microarray experiments (e.g. Affymetrix) are widely used, but their design and normalization procedures are not comparable with 2-channel experiments and were therefore not included in the study. See Table 1 for a summary of the data. Henceforth, we will refer to the data sets using the name of the first author of the corresponding publication i.e., the seven data sets are Alizadeh [13], Finak [14], Galland [15],Herschkowitz [16], Jones [17], Sørlie [18], and Ye [19].

**Table 1:** Data sets

Name	Description	Size**^1^**	Classes
Alizadeh [13]	Diffuse large B-cell lymphoma (DL-BCL) and other lymphoid malignancies (FL and CLL), normal cell samples as well as tissue type cell lines. http://llmpp.nih.gov/lymphoma, GEO: GSE60	133 × 7806 (133 × 7430)	DLCL (68) and other (65) samples (FL, CLL, normal cell samples and a variety of cell lines).

Finak [14]	Samples from epithelial and striomal tissue from breast reduction tissue from tumor-adjacent normal tissue. Agilent microarrays. GEO: GSE4823	66 × 33491	epithelial (34) and stromal tissue (32)

Galland [15]	40 non-functioning pituitary adenomas (NFPAs). Agilent microarrays. arrayExpress: E-TABM-899	40 × 40475 (40 × 40291)	invasive (22) and non-invasive (18)

Herschkowitz [16]	Human breast tumor samples (The full study includes 232 samples, but here only 119 samples run on a particular array (GPL1390) are included). GEO: GSE3165	106 × 19718	ER-status, ER+ (59) and ER- (47)

Jones [17]	High-grade lung neuroendocrine tumors. GEO: GSE1037	91 × 40233 (91 × 39746)	Patients with (72) and without (19) cancer.

Sørlie [18]	Human breast carcinomas. http://genome-www.stanford.edu/breast_cancer/mopo_clinical/data/mopo_clinical.gz.tar	73 × 8033 (73 × 7734)	Clinical ER-status ER+ (55), ER- (18)

Ye [19]	Samples from 40 hepatitis B-positive patients with hepatocellular carcinoma (HCC). GEO: GSE364	87 × 8911	metastatic (P) and non-metastatic (PN) patients. P (65), PN (22)

A summary of the data sets used in this study.

^1^Number of samples × Number of genes, the values are after duplicates have been joined and after the filtering with respect to missing values. For those data sets where the resulting dimension is not the same for the background corrected normalization (norm.pt.bkg and norm.glob.bkg) the dimension given in parenthesis is for norm.pt.bkg and norm.glob.bkg.

As the goal of this study is to investigate how the performance of various cluster analysis algorithms is affected by the choice of pre-processing and gene selection, we need a true class partitioning to compare the computed clusters with. One should note, however, that there are often more than one way to partition data and that "true classes" in this context is a relative term.

We have focused on data that can be divided into two distinct classes, so that the results are more comparable between data sets. For some data sets several alternative class partitionings are possible. Here is a short description of the data sets and the classes that are used for the results presented in this paper. See also Table 1 for a summary.

#### Ye [19]

This data set includes samples from patients with hepatocellular carcinoma (HCC). The survival of the patient is highly correlated with metastasis. In the original study [19] the difference between metastatic and non-metastatic patients is studied and this is also how we define our two classes; samples from patients with metastasis and samples from patients without metastasis.

#### Alizadeh [13]

This data set consists of samples from patients with diffuse large B-cell lymphoma (DLBCL) as well as samples from other lymphoid carcinomas (follicular lymphoma (FL) and chronic lymphocytic lymphoma (CLL), as well as normal cell samples and a variety of cell lines (see [13] for details)). The classes that we have used for this data are DLBCL and a class including all other samples.

#### Sørlie [18]

The purpose of the original study [18] was to classify breast carcinomas based on gene expression. Here, we want to use classes that are not defined by the gene expression, but rather by a clinical observation. In the hierarchical clustering in [18] the samples separate in two groups, one with low levels or no expression at all of ER and one with high ER expression. Therefore we decided to use classes based on the ER (estrogen receptor) status of the tumor sample i.e., the two classes are ER+ and ER-.

#### Herschkowitz [16]

This is another breast cancer data set and we decided to use the same classes as for Sørlie i.e., ER status. In the original study the gene expression data was used to define novel cancer subtypes.

#### Jones [17]

The paper [17] aims to classify high-grade neuroendocrine tumors (HGNT) of the lung and in the study a number of different carcinomas as well as normal lung samples are included. Here, we have focused on the two most distinctive classes in this data set, the cancerous and the non-cancerous samples.

#### Finak [14]

The original breast cancer study [14] investigate whether normal epithelium and stroma have distinct expression profiles compared to tumor-adjacent normal tissue using Agilent microarrays. The conclusions are that morphologically normal epithelium and stroma exhibited distinct expression profiles, but molecular signatures that distinguished breast reduction tissue from tumor-adjacent normal tissue are absent. The two classes that we use are defined by the distinguishable tissue types i.e., epithelial and stromal tissue.

#### Galland [15]

This data set consists of 40 non-functioning pituitary adenomas (NFPAs) classified as invasive or non-invasive on the basis of magnetic resonance imaging and surgical findings. The original study [15] used the Agilent microarray in order to identify genes with differential expression between invasive and non-invasive NFPAs. Here, we have used invasive and non-invasive NFPAs as our two classes.

All the data sets result from cDNA microarray experiments where a sample is compared to a common reference. The data values that we have been working on in this study are the log_2_-ratios (M-values) i.e., the logarithm with base 2 of the ratios between treated and reference channel.

### Cluster analysis

In this paper, 2780 cluster analysis approaches for cDNA-microarray data are considered. Here, a cluster analysis involves the consecutive sub-processes: normalization of the microarray data, imputation of missing values, gene selection, standardization and clustering. Within each sub-process several methods are considered. We have strived to include commonly used methods, but some rare methods have been included since they contrast the commonly used ones. The methods and the rationale for including them are described below.

#### Normalization

The methods considered in the normalization sub-process are the four combinations of two dye-normalization methods and two types of background correction. The two dye-normalization methods are global MA-loess [20] and print-tip (local) MA-loess [21]. Both methods estimate the dye effect by modeling the log_2_-ratios (M) as a function of the average log_2_-intensity (A) using local regression (loess). In the global MA-loess the dye effect is estimated on data from the entire array, while in the print-tip version the dye effect is estimated individually on smaller subsets of the array. Both the dye normalization methods were applied to either background corrected data or data without background correction. For the background correction the local correction method is used [22], where the background estimates obtained from the image analysis are subtracted from the foreground estimates.

In addition to the normalized data the raw data is included in the study. Henceforth the raw- and the normalized- data are denoted as; no.norm - no normalization; norm.pt - print-tip MA-loess; norm.pt.bkg - print-tip MA-loess with background correction; norm.global - global MA-loess and norm.global.bkg - global MA-loess with background correction.

The considered normalization methods are commonly used, although print-tip normalization without background correction is often recommended [23,24]. Background correction generally decreases the experiments ability to get unbiased estimates of the genes regulation (i.e. the bias), but increases the variance and consequently decreases the experiments sensitivity [25]. Studies of how the trade-off between bias and sensitivity affects the performance of the cluster analysis has to our knowledge not been done.

#### Filtration and missing value imputation

Spots flagged by the scanner or the experimentalist, and also spots with lower signal than background (when background correction is adopted) are marked as missing values, see e.g. [25] for a discussion on how to treat flagged spots. Only samples with less than 50% missing values and genes with less than 30% missing values are included (samples and genes are filtered simultaneously). The remaining missing values are imputed [8,26]. Here, we have adopted two commonly used approaches for missing value imputation;

**ROW **A missing value is replaced by the row median, i.e. the median for that gene.

**SVD **The R-function svdImpute in the package pcaMethods implements the SVDimpute algorithm as proposed by [26]. This algorithm estimates missing values as a linear combination of the *k *most significant eigengenes. SVD (singular value decomposition) is used to find the set of orthogonal principal components or eigengenes that will be used to estimate the entire data matrix (through linear combination). For a particular gene with missing values, regression is used to find the coefficients for the eigengenes. In the regression, missing values are removed (and the corresponding values in the eigengenes are of course also removed). The coefficients are then used to estimate the missing values by linearly combining the eigengenes. The method is iterative. In the first round all missing values are replaced by 0 when the eigengenes are computed, in the following rounds the missing values are replaced by their estimates. The iteration stops once the change in the data matrix between two iterations is small enough.

In some of the data sets the annotation of the samples is incomplete and samples with unknown class identity had to be removed. Finally, the data matrix is further reduced by computing the mean values for duplicate genes.

#### Gene selection

In most microarray analyses it is common to select a subset of genes prior to the analysis. The purpose of the gene selection can for example be to reduce the overall noise in the data or to be able to perform a particular downstream analysis. Not performing gene selection can be an alternative as well, although simulations in have indicated that keeping irrelevant genes during cluster analysis result in reduced accuracy [9].

Detecting the relevant genes is not trivial and we include several different approaches for gene selection in this study. Below follows a list of gene selection methods that are adopted in this study;

**STD **Select the *N *genes that have the highest standard deviation [27]. This is a widely used method, motivated by the fact that a differentially expressed gene will have M-values (log_2_-ratios) with a relative high standard deviation. However, the drawback is that variable genes may have high standard deviation although they are not differentially expressed.

**M **Select the *N *genes with highest absolute log_2_-ratios (M-values). This is a natural method to apply when the common reference design is used and two treatments are compared to a common reference. It is based on the assumption that few genes are differentially expressed and that it is unlikely that one treatment is up-regulated and the other down-regulated against the common reference.

Consequently, a high absolute M-value implies that the gene may separate the classes. However, there is a risk that both treatments are equally differentially expressed towards the common reference.

**T1 **Select the *N *genes with highest modified t-statistic;

t1=|M¯σM,modif2n|,

where $\bar{M}$ denotes the mean value of M, $\sigma_{M}^{2}{,}_{modif}$ is the variance of M (modified according to Baldi-Long [28]) and *n *is the number of samples. This method is to our knowledge rarely used, but we choose to include it for two reasons; it's a natural prolongation of the M-method and it contrasts the STD-method.

**PC **Principal component analysis (PCA) is performed on the data matrix and the *k *most significant principal components ("*eigengenes*") are used in the clustering procedure (instead of a subset of genes). This commonly used method makes a linear projection of the data into a low-dimensional subspace defined by a number of principal components that explain the most variance.

**NONE **No gene selection is done, hence all genes are included (due to computational costs this means that the model-based method (Mclust) is not used). This approach is surprisingly common, but previous studies have shown that clustering performance may be degraded when irrelevant genes are selected.

In all the above methods, except PC and NONE, the number of selected genes, *N*, has been set to 15, 100, and 1000 (Mclust is not run with 1000 genes). In PC, the number of principal components has been set to 3, 5, and 15. The numbers were selected at levels believed to cover what is reasonable in practical applications. For some analyses, additional levels were investigated.

In total this results in 13 unsuperwised gene selections, these will be referred to by their method abbreviation (as above) followed by a number referring to the number of selected genes or the number of principal components.

### Class dependent gene selection

We also apply two methods that use the class information in the gene selection i.e., that select genes that can separate the classes. It is intuitive to believe that a clustering analysis conducted on discriminatory genes will retrieve the classes that these genes discriminate between. However, it is important to point out that with a gene selection of this type the cluster analysis can no longer be considered as an unsupervised method [29]. We still include these methods as a positive control. The two methods are;

**T2 **Select the *N *genes with highest modified t-statistic comparing classes A and B,

$$t_{2}=\left|\frac{{\bar{M}}_{A}-{\bar{M}}_{B}}{\sqrt{\frac{\sigma_{M_{A},modif}^{2}}{n_{A}}+\frac{\sigma_{M_{B},modif}^{2}}{n_{B}}}}\right|.$$

Again the modified variances are approximated according to Baldi-Long [28].

**Mdiff **Select the *N *genes with the largest difference in M between classes A and B, i.e. $\left|{\bar{M}}_{A}-{\bar{M}}_{B}\right|$.

#### Standardization

Standardization is a technique to put equal weight to the genes and is commonly used when the cluster analysis is based on expression levels. However, for the common reference design the analysis is based on log_2_-ratios. We have included standardization using the Z-transformation [30], i.e. scaling of the genes to mean 0 and standard deviation 1. In addition, we have considered non-standardized data. The normalization, missing value imputation and gene selection are performed before the potential standardization.

#### Clustering

We have applied five commonly used types of clustering methods. Some of these methods have several different settings and taking a carefully selected subset of these into account resulted in 11 different clustering methods.

**hclust **The agglomerative hierarchical cluster analysis included in this study is implemented in the R-function hclust included in the package stats. The clustering is based on a set of dissimilarities between the samples. Here we have used dissimilarities based on the Euclidean distance, the Manhattan distance or the Pearson correlation coefficient (transformed into a distance by taking 1 minus the correlation coefficient). In addition, we have used two different methods for computing similarities between clusters; Ward's method [31] and the average linkage (UPGMA) method [32]. We will refer to the hierarchical clusterings as hclust.dist.meth, where dist and meth are unambiguous acronyms for the distance measures (eucl, manh, corr) and agglomeration methods (ward, ave), respectively.

**kmeans **The k-means clustering algorithm that we use here is implemented in the R-function kmeans (package: stats). In this function, 100 random starting sets (sets of *k *cluster centers) are used and the algorithm adopted is that of Hartigan and Wong [33].

**PAM **Partitioning of data into *k *clusters around medoids (representative objects) can be done using the R-function pam in the package cluster. We have adopted two variants of defining distances in the function; Euclidean distance and Pearson's correlation.

**SOM **(Self-organizing maps). SOMs represent multidimensional data in a low-dimensional topological map. The grid used here is one-dimensional and the number of grid points equals the number of clusters. The implementation of SOM in the R-function som in the package kohonen [34] is used.

**Mclust **We employed model-based clustering using expectation maximization initialized by hierarchical clustering for parametrized Gaussian mixture models [35]. Here, the R-function Mclust in the package mclust is used. Due to computational costs, Mclust is only adopted after gene selection reducing the data to 500 genes or less.

### Cluster evaluation

There are more than one way to evaluate the quality of a clustering, both when the true class identities are known and when they are not. Measures of clustering quality that are independent of the true partitioning include the average silhouette width (asw) and the Calinski-Harabasz (CH) index [36,37]. Although the focus here is on measures that compare true classes to a predicted clustering, measure such as asw and CH can also be of interest e.g., to determine the optimal number of clusters.

For all the data sets considered in this study the true number of clusters is two. However, to fix the number of predicted clusters to two might not be optimal. There can for example be a few outliers that are placed in clusters of their own, forcing the two interesting clusters to be merged. In a manual evaluation this would be trivial to detect. In an attempt to mimic a visual inspection of the clusters we have come up with the following procedure for cluster evaluation; Instead of two, we predict ten clusters. The clusters are then joined into two clusters in an optimal way by optimizing a similarity score comparing the true partitioning with the joined predicted partitioning.

In this study we use the adjusted Rand index (aRand) as a measure of performance, since this is a sensitive measure recommended in the literature [38,39].

#### Rand and adjusted Rand index

The Rand index [40] measures the agreement between two partitionings, *P *= {*P*_1_, ..., *P_s_*} and *Q *= {*Q*_1_, ..., *Q_t_*}, of a data set of *n *objects *x*_1_, *x*_2_, ..., *x_n _*with respect to every pair of objects. The Rand index is the fraction of object pairs that either are in the same cluster in both partitionings or in different clusters in both partitionings. Formally, the Rand index is defined as;

$$Rand:=\frac{a+b}{a+b+c+d}=\frac{a+b}{\left(\begin{array}{c} n\\ 2 \end{array}\right)},$$

Where

$$\begin{array}{l} \;\begin{array}{cc} a=|A|, & \text{where }A=\{(x_{i},x_{j})|x_{i},x_{j}\in P_{k};x_{i},x_{j}\in Q_{l}\} \end{array}\\ \begin{array}{cc} b=\;|B|, & \text{where}\;B=\{(x_{i},x_{j})|x_{i}\in P_{k_{1}},x_{j}\in P_{k_{2}};x_{i}\in Q_{l_{1}},x_{j}\in Q_{l_{2}}\}, \end{array}\\ \begin{array}{cc} c=\;|C|, & \text{where}\;C=\{(x_{i},x_{j})|x_{i}\in P_{k_{1}},x_{j}\in P_{k_{2}};x_{i},x_{j}\in Q_{l}\}\text{ and} \end{array}\\ \begin{array}{cc} d=\;|D|, & \text{where}\;D=\{(x_{i},x_{j})|x_{i},x_{j}\in P_{k};x_{i}\in Q_{l_{1}},x_{j}\in Q_{l_{2}}\}, \end{array} \end{array}$$

for some *k*, *k*_1_, *k*_2 _∈ {1, ..., *s*} and *l*, *l*_1_, *l*_2 _∈ {1, ..., *t*}, where *k*_1 _≠ *k*_2 _and *l*_1 _≠ *l*_2_.

The Rand index is a number between 0 and 1. The adjusted Rand index is more commonly used as it has an expected value of zero (for random partitioning), whereas the expected value of the Rand index varies. The adjusted Rand index (sometimes called the corrected Rand index) is defined as;

aRand:=Rand−E[Rand]max(Rand)−E[Rand],

Where *E*[ ] denotes the expected value. aRand is a number between -1 and 1.

Since we compute aRand after joining ten initial clusters into two by optimizing aRand, the expected value of aRand will be greater than 0. By simulating 1000 random partitionings (of ten classes) and computing optimal aRand in the same way, we can estimate the expected value and the distribution of aRand by chance. The expected value varies a bit between data sets as the true classes vary both in total number of samples and relative class sizes.

## Results and Discussion

In this study we have included seven data sets and a large number of cluster analysis approaches (normalization, standardization, gene selection, missing value imputation, clustering method). In total we have performed 2780 analyses per data set (not counting the 1280 cluster analyses where the gene selection depends on the true classes). All analyses are applied to the seven data sets resulting in 19460 cluster analysis that are all compared to the true partitioning using a cluster evaluation score (see previous section).

The gene selection methods T2 and Mdiff use class informations to select genes and are included as a positive control (i.e. we can not expect gene selection methods that don't use the class information to do better than this), but we will not include the result from these selections methods in our general conclusions. If not stated specifically, the results presented in the text and figures in this section are based only on the gene selection methods STD, M, T1, PC and NONE.

### Differences between the data sets

**Figure 1:**
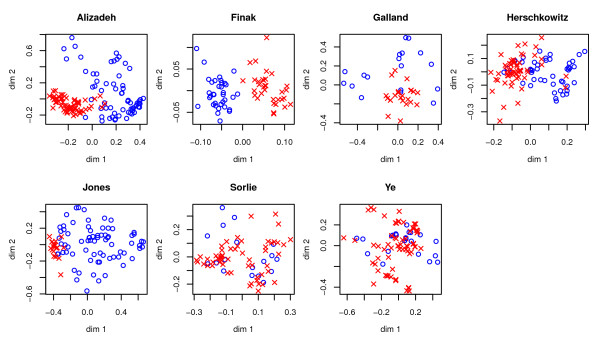
**Multidimensional scaling to visualize classes**. Scatter plots visualizing the two classes in each of the data sets. Multidimensional scaling was performed on the correlation based distance matrices producing scatter plots that try to preserve the distances between observations. The two classes are plotted as either red diamonds and blue circles, corresponding for each data set to the classes; Alizadeh: DLCL and other, Finak: epithelial and stromal tissue; Galland non-invasive and invasive; Herschkowitz: ER+ and ER-, Jones: cancer and not cancer, Sørlie: ER+ and ER-, and Ye: P and PN. These figures illustrate how easy it is to separate the classes (in two dimensions) e.g., Alizadeh have quite well separated classes, whereas Sørlie have two classes that are not easily separated.

**Figure 2:**
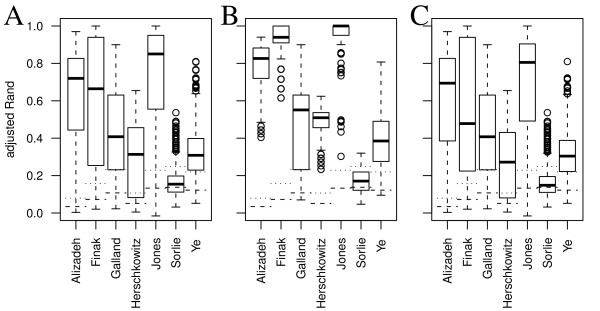
**Difference between data sets**. Boxplots showing the aRand values for the five data sets. A. The distribution of aRand values for each data set over all 2780 cluster analysis approaches. B. The same distribution, but based only on the 288 cluster analyses that remained after the reduction of cluster analyses. C. The complement to B i.e., distributions based on the 2492 cluster analyses that were removed. The distribution of aRand values for random classifications for each data set is indicated in the plots by horizontal lines representing the median and 95-percentile of the aRand values (dashed and dotted, respectively).

The performance varies considerably between the data sets. This is expected as the true class definitions vary between the data sets and agreement between these classes and the expression patterns differ. Figure 1 show scatter plots of the two classes in each data sets by multidimensional scaling [41] of correlation based distance matrices. Figure 2A shows boxplots of the distribution of aRand values for each of the seven data sets for all the 2780 cluster analyses. Jones, with the classes cancer and not cancer, has a very high median aRand value, whereas the data sets where metastasis or ER-status define the classes show lower aRand values. Also Alizadeh has a high median aRand value. Here the classes are DLCL (a type of lymphatic cancer) and other (including FL and CLL (two types of lymphatic cancer), normal cells as well as a variety of cell lines). The difficulty of separating some of the data sets into the two defined classes can to some extent be seen in Figure 1 and corresponds relatively well with the actual clustering performance in Figure 2A.

The performance of the clustering methods on the Sørlie data set is very close to what is expected by chance, indicating that the samples do not separate into ER- and ER+ based on the expression data. Henceforth, the Sørlie data set will therefore be excluded from this study.

### Overall influence of the sub-processes

To see the influence of the sub-processes, i.e. normalization, standardization, gene selection, missing value imputation and clustering method, we build a regression model using the aRand value as response variable and the sub-processes, as well as the choice of data set, as predictor variables. Also interaction terms of order two are included in the model. (The model is computed in R by the function lm and an analysis of variance table is computed using the R-function anova in the package stats).

The result shows that the data set is most important, explaining 26.5% of the total variability in the response variable. Second most important is the gene selection (including the number of genes to select) (15.0%), followed by clustering method (4.9%), normalization (0.66%), and standardization (0.01%). All of these variables have a significant influence on aRand (*p <*0.001), whereas missing value imputation method is of minor importance.

**Figure 3:**
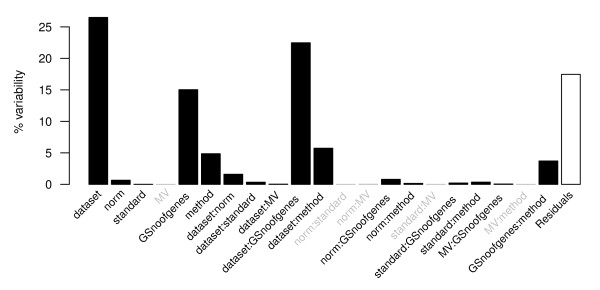
**Regression model**. The percent variance explained by each of the variables and their cross terms (second-order interactions). The black bars show significant terms (*p *< 0.001). The variables are the following: data set - the six data sets Alizadeh, Finak, Galland, Herschkowitz, Jones and Ye; norm - the five normalizations no.norm, norm.pt, norm.pt.bkg, norm.glob and norm.glob.bkg; standard - standardization or not; MV - missing value imputation using one of the methods ROW or SVD; GSnoofgenes - gene selection method and number of selected genes (in total 13 variants); method - the clustering methods hierarchical clustering (six different settings), k-means, PAM (two variants), SOM and Mclust. A cross term between A and B is denoted A:B. The percent variability explained by the residuals is the variability not explained by the regression model.

The results also show that the influence of the interaction terms are quite strong (Figure 3), suggesting that different sub-processes interact. Notably all second order interactions with data set are significant, suggesting that the performances of all sub-processes vary between the data sets. Due to the variability between the data sets we mainly focus on showing the effects of interactions between data sets and clustering method, normalization and gene selection. Although it would be interesting investigate the interactions between any and all sub-processes, the number of possible combinations are to numerous to be completelty covered in this study. number of dataset woud have to be significantly increased). The results of this study can however be used as a guideline for choosing specific combinations. Therefore the data and an example of how it can be used to investigate choices of methods are included in Additional files 1, 2, 3, 4, 5, 6.

### Reduction of the number of analyses

In an attempt to find a robust and high performing cluster analysis, pair wise method comparisons within the most important sub-processes (normalization, gene selection method and clustering method; see Figure 3) are made to remove non-favorable methods settings (e.g. within clustering methods, kmeans is compared to SOM etc.). The following iterative process for removing methods with relativly low performances is considered:

• Within each sub-process all methods are compared pair wise with respect to the adjusted Rand using Wilcoxon's signed rank test. The resulting p-values are corrected using Bonferroni's correction (i.e. multiplied by the total number of tests).

• The pair of methods generating the minimum p-value is identified and if the corrected p-value is below 0.001 the pair's least favorable method is considered to be a non-favorable method.

• All cluster analyses involving the non-favorable method are removed and the process continued until no more removals are made.

The process removes methods that have superior competitors. The process, presented in Table 2, reduced the initial 2780 analyses to 288 analyses defined by the sub-processes: normalization (norm.pt, norm.pt.bkg, norm.glob, norm.glob.bkg), standardization (notstd, std), MV (ROW, SVD), gene selection (NONE, STD 100, STD 1000, and PC 15) and clustering method (hclust.corr.ward, hclust.eucl.ward, hclust.manh.ward, kmeans, Mclust).

**Table 2:** Parameter filtering

Removed method	p-value	Superior method
T1 15	9.468e-207	PC 5
T1 100	4.037e-127	PC 5
M 15	1.435e-111	PC 5
hclusteuclaverage	4.926e-100	kmeans
hclustmanhaverage	4.536e-97	kmeans
STD 15	1.556e-88	STD 1000
M 100	2.045e-76	M 1000
P0	9.134e-75	P1
PC 3	1.241e-50	PC 15
T1 1000	3.739e-36	PC 15
pameucl	4.036e-32	kmeans
PC 5	1.033e-21	PC 15
pamcorr	8.688e-18	hclustcorrward
som	1.340e-13	kmeans
hclustcorraverage	2.337e-10	hclustcorrward
M 1000	4.359e-07	PC 15

In an iterative process pairwise comparisons of normalization, gene selection and clustering methods are made to remove less favorable methods. This table presents the removed methods, the uncorrected p-value resulting from a Wilcoxon one-sided test comparing the removed method with a superior method.

As can be seen in Figure 2B the distribution of aRand values for each data set after the above filtration are generally shifted towards higher aRand values (see also Figure 2C for the complement to the selected approaches).

This initial reduction process is used to state the following hypotheses:

• Normalization of data is likely to increase the performance of cluster analyses.

• The number of genes (or principal components) should be relatively high.

• Gene selection methods using the adjusted t-statistic (T1) and the mean of the M-value (M) are likely to perform worse than the STD and PC selection methods.

• Hierarchical clustering using Ward's method for calculating the distance between clusters is likely to perform better than analyses using average linkage.

The above hypotheses are further investigated by considering more detailed comparisons, based on the sub-processes that remained after the initial filtering unless otherwise stated.

In the following analyses all methods within a sub-process are compared against each other. In total 78, 55 and 10 pairwise comparisons are made for the unsupervised gene selection, clustering and normalization sub-processes, respectively. Two methods are considered significantly different if the Bonferroni corrected p-value is less than 0.01.

### Gene selection

**Figure 4:**
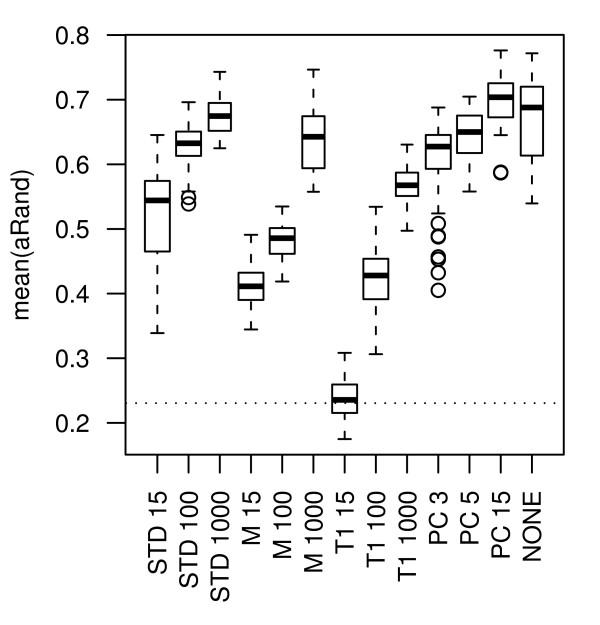
**Gene selection methods**. Boxplots showing the mean aRand (mean taken over the data sets) for the gene selection methods and number of selected genes. The distributions represented by the boxplots are based on 80 (64) cluster analyses for the gene selections choosing 100 genes or less (number in parenthesis is for 1000 genes or more). The cluster analyses consist of combinations of the following sub-processes: normalizations norm.pt, norm.pt.bkg, norm.glob and norm.glob.bkg; standardization and nor standardization; missing value imputation by ROW and SVD; clustering methods hclust.corr.ward, hclust.eucl.ward, hclust.manh.ward, kmeans and Mclust (for 100 genes or less). The horizontal line shows the 95-percentile (dotted line) for the distribution of aRand values for random classifications (the median is outside the range of this plot).

In general, the performance is high when a relatively large number of genes is selected. For all gene selections there is a significant improvement in adjusted Rand between 15 and 100 genes and between 100 and 1000 genes. Interestingly, there is not a significant improvement by doing a gene selection compared to including all genes for any gene selection method. The only gene selections that do not significantly lower the performance compared to no gene selection are STD 100, STD 1000, M 1000, PC 5 and PC 15. See Figure 4 for a summary of the results.

STD and PC are the gene selection methods that perform best and STD perform significantly better than both M and T1 independently, regardless of how many genes that are selected.

**Figure 5:**
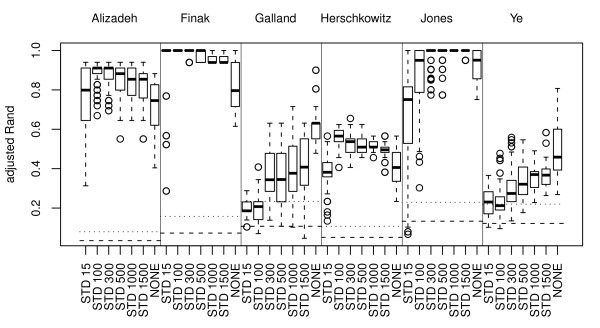
**Number of selected genes**. The figure shows details on how the number of selected genes affect aRand for the variance-based gene selection method STD for each of the six data sets Alizadeh, Finak, Galland, Herschkowitz, Jones and Ye. The distributions represented by the boxplots are based on 80 (64) cluster analyses for the gene selections choosing 500 genes or less (number in parenthesis is for 1000 genes or more). The cluster analyses consist of combinations of the following sub-processes: normalizations norm.pt, norm.pt.bkg, norm.glob and norm.glob.bkg; standardization and nor standardization; missing value imputation by ROW and SVD; clustering methods hclust.corr.ward, hclust.eucl.ward, hclust.manh.ward, kmeans and Mclust (for 500 genes or less). The horizontal lines show the median (dashed line) and 95-percentile (dotted line) for the distribution of aRand values for random classifications.

To further investigate how the number of genes affect the performance, we decided to also include a selection of 300, 500 and 1500 genes by STD. For four of the data sets (Alizadeh, Finak, Herschkowitz and Jones) the optimal performances are seen in the interval 100-1000 genes and for these optimal selections the performances are significantly better than including all genes (*p *< 0.01 after Bonferroni's correction, 21 tests). However, for the data sets Galland and Ye this is not the case, see Figure 5.

**Figure 6:**
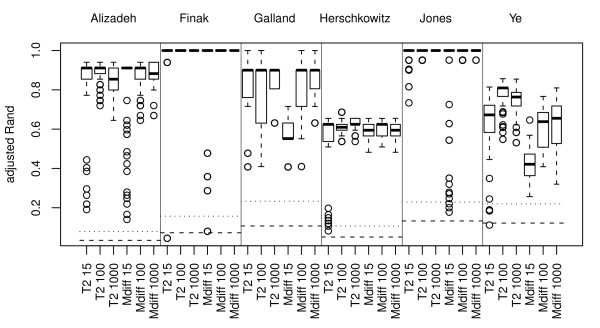
**Gene selection methods using class information**. The two supervised gene selection methods T2 (t-statistic) and Mdiff (difference in log_2_-ratios) try to identify genes that are differentially expressed between the two (known) classes. The boxplots in this figure show the mean adjusted Rand values between the clusterings computed after gene selection using T2 or Mdiff. The distributions represented by the boxplots are based on 80 (64) cluster analyses for the gene selections choosing 100 genes or less (number in parenthesis is for 1000 genes or more). The cluster analyses consist of combinations of the following sub-processes: normalizations norm.pt, norm.pt.bkg, norm.glob and norm.glob.bkg; standardization and nor standardization; missing value imputation by ROW and SVD; clustering methods hclust.corr.ward, hclust.eucl.ward, hclust.manh.ward, kmeans and Mclust (for 100 genes or less). The horizontal lines show the median (dashed line) and 95-percentile (dotted line) for the distribution of aRand values for random classifications.

Mdiff and T2 are supervised gene selections and give, as expected, significantly better results than the other gene selection methods and no gene selection, see Figure 6. Note that these methods perform rather good when just 15 genes are selected.

### Clustering methods

**Figure 7:**
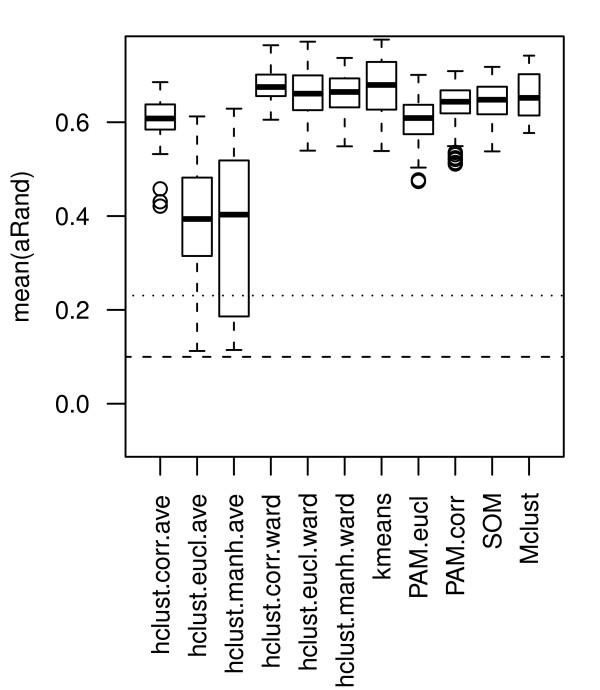
**Clustering methods**. Boxplots comparing the mean(aRand) values between clusering methods. The distributions represented by the boxplots are based on 64 (32) cluster analyses for each clustering method (number in parenthesis for Mclust). The cluster analyses consist of combinations of the following sub-processes: normalizations norm.pt, norm.pt.bkg, norm.glob and norm.glob.bkg; standardization and nor standardization; missing value imputation by ROW and SVD; gene selections STD 100, STD 1000 and PC 15). The horizontal lines show the median (dashed line) and 95-percentile (dotted line) for the distribution of aRand values for random classifications.

For the hierarchical clustering we employ six different variants using the distance measures; Euclidean, Manhattan and Pearson's correlation (1-correlation) and the linkage methods average and Ward. The most striking observation is that Ward's method performs significantly (adjusted *p <*0.01) better than average linking for all distance measures, see Figure 7. This is an observation that holds also for the data sets considered separately (except for the correlation distance), data not shown.

Hierarchical clustering using Ward's method and the correlation distance and k-means perform significantly better than PAM and SOM, see Figure 7. Note that Mclust cannot be directly compared to other methods, since there are fewer data points to compare for Mclust as only gene selections with up to 100 genes are included. However, based on the analyses where genes are selected using PC5, PC 15, and STD 100, Mclust performs significantly better than PAM.eucl, hclust.eucl.ave, and hclust.manh.ave, but significantly worse than kmeans.

### Normalizations

Five normalization procedures are compared i.e., 10 pair wise comparisons are made.

**Figure 8:**
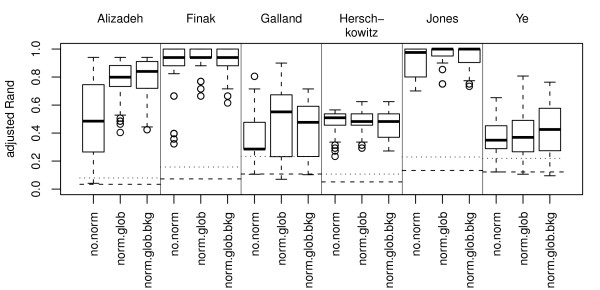
**Normalization**. Boxplots showing the performance (adjusted Rand) for the normalizations. The plot shows the distributions of aRand values for the data sets separately. supervised gene selection, selecting 100 genes using the methods Mdiff and T2. The distributions represented by the boxplots are based on 72 cluster analysis for each normalization.gene selections). The cluster analyses consist of combinations of the following sub-processes: standardization and not standardization; missing value imputation by ROW and SVD; gene selections NONE, STD 100, STD 1000 and PC 15; clustering methods hclust.corr.ward, hclust.eucl.ward, hclust.manh.ward, kmeans and Mclust (for 100 genes or less). The horizontal lines show the median (dashed line) and 95-percentile (dotted line) for the distribution of aRand values for random classifications.

As expected, normalization has a large positive impact on some data sets, in particular Alizadeh, Galland and Jones, but has only a limited effect on the other data sets, see Figure 8. This somewhat surprising result can to some degree be explained by the particular design of the experiments (the common reference design). Never the less, omitting normalization can have a large negative effect on the cluster analysis.

We will concentrate on the data sets that benefit most from normalization, i.e. Alizadeh, Galland and Jones. The Alizadeh and Jones data sets used custom made arrays and here both global and print-tip normalization are applied. We cannot see any significant (adjusted *p *< 0.01) differences between these methods (data not shown) and choose to concentrate on the global method since the print-tip method was not used on the Agilent data sets.

The choice of using or not using background correction has a clear impact on the performance, but there is no significant difference, see Figure 8. Arguably, methods using background correction have an increased variance for the observed aRand. This indicates that background correction works well in combination with some of the considered methods, but considerable worse with others. We argue that methods that are able to omit genes that cannot distinguish the true classes will work relatively well together with background correction. The reason for this is that background correction reduces the bias of affected genes (i.e. genes that can be used to separate the classes) and increases the variance of all genes [25]. The decrease in bias will improve the clustering, but the increase in variance will make gene selection more difficult and have a negative effect on the clustering. Hence, background correction should work relatively well if we only include a limited number of irrelevant genes. In order to test our hypothesis we perform gene selection using the supervised gene selection methods T2 and Mdiff with 100 genes. For the Jones data almost all analyses have aRand close to one and it is not possible to see any difference between the normalization methods, data not shown. Also for the Galland data there is no significant difference between the normalization methods. However, for the Alizadeh data the positive background correction effect (although non-significant) indicated using unsupervised gene selection become more evident after supervised gene selection (significant (adjusted *p <*0.01, 10 tests)).

## Conclusions

The optimal pre-processing and gene selection procedure is highly dependent on the data set subjected to the analysis and it is important to remember that the true classes used in this study are not the only alternative for dividing the data samples into classes. It is however clear that the choice of pre-processing, gene selection and clustering method does have an influence on the cluster analysis result. We also show that standardization and the choice of missing value imputation has a minor influence on the clustering. Of course missing value imputation as such is necessary, but the two methods (ROW and SVD) that we have adopted perform approximately equally well.

Furthermore, we show that normalization increases the performance and that there are some indications that background correction has a positive effect if a large proportion of the irrelevant genes can be filtered during gene selection.

Gene selection itself has a huge impact on the downstream cluster analysis and both the selection method and the number of selected genes are important. We show that gene selection does improve the results, but the number of selected genes need to be relatively high when using an unsupervised gene selection method. In a comparison between gene selection methods, the best alternatives are the standard deviation method and the principal component method. Furthermore, hierarchical clustering using Ward, k-means clustering and Mclust are the clustering methods that perform best in this study.

In this study the performance of over thousand cluster analysis methods are evaluated. We have focused on the more general effects; but there are clear indications that interactions between sub-processes play a vital role. As an example, consider a scenario where the user has decided to use k-means clustering on the 100 genes with highest standard deviation post standardization and imputation with row median, but are unsure of which normalization to apply. In this case Additional file 5 or 6 can be used to compare normalization procedures. For the above example the mean adjusted Rand index ranges from 0.61-0.69, with favor of normalization with background correction. Although it is not advisable to consider such an investigation as a general result it may provide guidelines to favorable method choices.

The study shows a high variability in the performance between the data sets. Consequently, there is a risk that the results are not valid for all experimental data. It should also be noted that our study focus on 2-channel experiments with a common reference design and that the conclusions cannot necessarily be extended to other platforms and experimental designs.

In summary, although clustering, gene selection and normalization are considered standard methods in bioinformatics, our comprehensive analysis shows that selecting the right methods, and the right combinations of methods, is far from trivial and that much is still unexplored in what is considered to be the most basic analysis of genomic data.

## Authors' contributions

EF was involved in developing the ideas in this paper, collecting the data sets, implementing the analysis, interpreting the results, and drafted the manuscript. ML implemented and conducted the normalizations of the data. JÖ examined the data sets and contributed in the general discussions. TRH helped collecting the data sets and contributed in the general discussions. PR was involved in developing the ideas presented in this paper and interpreting the results. All authors read and approved the final manuscript.

## Supplementary Material

Additional file 1 **Description and example of the Additional files**. A document describing all the additional files and an example of how these can be used to investigate choices of analysis methods. Click here for file

Additional file 2 **Description of the contents in the data files**. An excel document describing the contents of the data files in Addtional files 3-6. Click here for file

Additional file 3 **Adjusted Rand index**. A tab separated text file with adjusted Rand index for the seven datasets for each combination of parameters. Click here for file

Additional file 4 **Adjusted Rand index**. An excel file with adjusted Rand index for the seven datasets for each combination of parameters. Click here for file

Additional file 5 **Mean adjusted Rand index**. A tab separated text file with mean adjusted Rand index for each combination of parameters, the mean is taken over the six datasets (Sørlie is not included). Click here for file

Additional file 6 **Mean adjusted Rand index**. An excel file with mean adjusted Rand index for each combination of parameters, the mean is taken over the six datasets (Sørlie is not included). Click here for file
