# Supplementary material for: Challenges in microarray class discovery: a comprehensive examination of normalization, gene selection and clustering
Source: BMC Bioinformatics. 2010 Oct 11;11:503. doi: 10.1186/1471-2105-11-503 (PMC3098084; doi:10.1186/1471-2105-11-503)
Supplement: Additional file 1 — Description and example of the Additional files. A document describing all the additional files and an example of how these can be used to investigate choices of analysis methods. [file 1471-2105-11-503-S1.DOC]

Adjusted rand index and the mean adjusted rand index over six datasets for each combination of parameters are supplied in a tab separated text file and as an excel file. A description of each column of the two files are provided in an excel file.

As examples of how the supplementary can be used to investigate specific combination, we consider a scenario where the user has decided all parameters save which normalization to use.

Setting the parameters to k-means, STD 100, standardize and ROW imputation show that the difference in mean adjusted rand index as choice in normalization ranges from 0.61-0.69 with favor of background correction though little impact global or print-tip normalization.

| Normalization | Mean adjusted rand index |
| --- | --- |
| no.norm | 0,62 |
| norm.pt | 0,63 |
| norm.pt.bkg | 0,69 |
| norm.glob | 0,61 |
| norm.glob.bkg | 0,68 |
